# Early Sitting Improves the Accuracy of Predicting In-Hospital Mortality in Older Adult Patients With Pneumonia

**DOI:** 10.7759/cureus.84952

**Published:** 2025-05-28

**Authors:** Hidenori Akaiwa, Yosuke Morimoto, Ryuichiro Takamura, Kyoko Tanaka, Takako Matsubara

**Affiliations:** 1 Faculty of Rehabilitation, Kobe Gakuin University Graduate School, Kobe, JPN; 2 Department of Rehabilitation, Baba Memorial Hospital, Sakai, JPN; 3 Department of Respiratory Medicine, Baba Memorial Hospital, Sakai, JPN

**Keywords:** in-hospital mortality, older adults, physical therapy, pneumonia, prognostic factor, sitting

## Abstract

Introduction

Older adult patients with pneumonia have high in-hospital mortality rates, and their clinical course is complex and varied. Consequently, determining whether to prioritize early mobilization or palliative care by physical therapists is challenging. Therefore, this study aimed to investigate whether early sitting increases the accuracy of predicting in-hospital mortality among older adult patients with pneumonia.

Methodology

This was a single-center retrospective cohort study. We included patients aged ≥65 years who were hospitalized with pneumonia. Early sitting was defined as Intensive Care Unit Mobility Scale (IMS) category 3 within a week of admission. Four multiple logistic regression models were constructed to evaluate the influence of early sitting or not as a predictor of in-hospital mortality, with factors at admission based on a literature review. To evaluate the contribution of early sitting to the discriminatory performance of each logistic regression model, we compared the area under the curve (AUC) of each model with and without the inclusion of this variable using DeLong’s test for correlated receiver operating characteristic (ROC) curves.

Results

Of the 348 patients, 47 (13.5%) and 301 (86.5%) were nonsurvivors and survivors, respectively. All models identified early sitting as a predictor of in-hospital mortality (model 1: odds ratio [OR] = 0.09, 95% confidence interval [CI], 0.04-0.22, *P* < 0.001; model 2: OR = 0.06, 95% CI, 0.02-0.13, *P* < 0.001; model 3: OR = 0.07, 95% CI, 0.03-0.15, *P* < 0.001; model 4: OR = 0.06, 95% CI, 0.03-0.14, *P* < 0.001). The AUC increased in all models when early sitting was added as a factor compared with and without that (AUC of model 1, 0.819 vs. 0.874, *P* = 0.005; model 2, 0.713 vs. 0.831, *P* < 0.001; model 3, 0.733 vs. 0.820, *P* < 0.001; model 4, 0.709 vs. 0.817, *P* < 0.001).

Conclusions

It is suggested that the addition of early sitting by a physical therapist as a post-hospitalization process to the previously reported in-hospital mortality factors for older adult patients with pneumonia may improve their predictive accuracy.

## Introduction

Pneumonia is a leading cause of infectious disease-related mortality worldwide [1], with morbidity and mortality increasing with age [2]. In Japan, a super-aged society, pneumonia is the fifth leading cause of death, and 95% of these deaths occur in patients aged ≥65 years [3]. Moreover, the mortality of older adult patients with pneumonia is anticipated to increase. Previous studies have identified older age, sex, and body mass index (BMI) as the main predictors. Then, multimorbidity, disease severity, the geriatric nutritional risk index (GNRI), and functional status were also reported as predictors of mortality in patients with pneumonia [4-7]. However, in recent years, there has been an increase in older adult patients with pneumonia who are characterized by decreased physical function and dysphagia due to comorbidities and frailty [8]. These characteristics frequently lead to complex treatment plans and varied progress during hospitalization. Consequently, predicting mortality in older adult patients with pneumonia based on factors at admission alone may be challenging [9].

A scoping review has identified several aspects of clinical management in older adult patients with pneumonia, including not only diagnosis and treatment, but also nutrition, oral management, rehabilitation, prognosis, decision making, and palliative care [10].

In rehabilitation, early mobilization is recommended for patients with pneumonia who are at risk of physical decline and have difficulty returning home after hospitalization [8]. In some high-risk cases, treatment strategies may be modified depending on the patient’s clinical course, and a transition to palliative care, which prioritizes comfort, may be considered when appropriate, requiring careful and individualized decision-making [11]. Considering this perspective, early mobilization, which can be physically stressful, is not routinely emphasized in palliative care, where the goal is to enhance patient comfort rather than functional recovery. Therefore, more accurate predictors of in-hospital mortality in older adult patients with pneumonia should be identified. Physical therapists determine the suitability for early mobilization using established guidelines and subjective assessments; however, the possibility of palliative care can be a concern in treatment selection. In a different light, the physical therapist’s ability to objectively and subjectively assess a patient’s condition and make a comprehensive decision about whether or not to perform early mobilization may reflect the patient’s clinical status during the early post-admission phase. These assessments may complement the limitations of relying solely on admission-based prognostic factors. In particular, sitting is an important turning point in early mobilization.

Therefore, this study aimed to investigate the accuracy of prognostic factors for in-hospital mortality in older adult patients with pneumonia, focusing on the early sitting following hospitalization.

## Materials and methods

Study design and population

This was a single-center retrospective cohort study. Consecutive patients who were admitted to Baba Memorial Hospital, Sakai, Japan, with a diagnosis of pneumonia from April 1, 2022, to September 30, 2023, were enrolled. As per the Japanese Respiratory Society Management of Pneumonia guidelines in adults [12], the diagnosis of pneumonia was based on the presence of lower respiratory tract symptoms (e.g., cough, sputum, dyspnea, and chest pain) and systemic symptoms (e.g., fever, headache, muscle pain, joint pain, and psychological symptoms) and was confirmed by new infiltration on chest X-rays or computed tomography. In our hospital, the diagnosis of pneumonia at admission was made by the attending physician of the primary department, which was determined based on the patient's presenting condition. Departments included respiratory medicine, neurology, cardiology, gastroenterology, and others. When clinical symptoms and imaging findings met the diagnostic criteria for pneumonia, it was recorded as the principal diagnosis, regardless of the department. In some cases, the primary department was subsequently changed to respiratory medicine for further management. The exclusion criteria were patients aged <65 years, nonprescription of rehabilitation, lack of medical records, or missing data.

Ethical declarations

This study was approved by the Institutional Ethics Committee of Baba Memorial Hospital (approval number: 2023-50) and Kobe Gakuin University (approval number: 24-01). An opt-out option was provided, allowing patients to withdraw from the study at their discretion. All data were de-identified before analysis, and electronic files were stored on an encrypted, password-protected server accessible only to the research team. Those who were rejected were excluded. This study was conducted according to the Declaration of Helsinki.

Measures 

In-hospital mortality was the primary outcome, and several variables were compared between the survivor and nonsurvivor groups who died during hospitalization. Basic participant characteristics at admission and clinical course, including the day of the first sitting by physical therapists, were collected. Data on deaths during hospitalization were collected from electronic medical records, and the in-hospital mortality rate was calculated.

Basic characteristics at admission encompassed diagnosis, age, sex, and blood test results (white blood cell count [WBC], C-reactive protein [CRP], serum albumin [ALB], and blood urea nitrogen [BUN]). The type of pneumonia was classified as either community-acquired pneumonia (CAP) or healthcare-associated pneumonia (HCAP). HCAP was defined as pneumonia in patients with any of the following conditions: hospitalization during the preceding 90 days; residence in a nursing home; or immunosuppressed conditions, including cancer, immune disorders, steroid use, immunosuppressant use, and chronic dialysis based on the American Thoracic Society and Infectious Diseases Society of America [13]. Patients not meeting the criteria for HCAP were classified as CAP. Pneumonia severity was categorized as A-DROP, which is the modified version of the CURB-65 score proposed by the Japanese Respiratory Society [12]. The A-DROP score, which includes age ≥70 years for males or ≥75 years for females, blood urea nitrogen (BUN) ≥21 mg/dL or signs of dehydration, oxygen saturation ≤90% by pulse oximetry or arterial oxygen partial pressure ≤60 mmHg, confusion, and systolic blood pressure ≤90 mmHg, was calculated by assigning one point for each applicable criterion, with higher scores indicating greater disease severity [12]. The Charlson comorbidity index (CCI) was employed for evaluating comorbid conditions. The CCI [14] is a weighted index of multiple comorbid conditions and encompasses cerebrovascular disease, diabetes with or without chronic complications, congestive heart failure, myocardial infarction, kidney disease, liver disease, chronic lung disease, dementia, hemiplegia or paraplegia, malignant tumors, leukemia, collagen disease, lymphoma, and human immunodeficiency virus and acquired immunodeficiency syndrome. Each variable is assigned a weight (0, 1, 2, 3, 4, or 5 or more), and the CCI score is represented as a total score, with a higher score indicating a greater number of comorbidities. Nutritional status was assessed by BMI. GNRI [15], which is reported as a predictor of in-hospital mortality, was calculated from the ALB and BMI values obtained on hospital admission using the following formula: GNRI = 14.89 × ALB (g/dL) + 41.7 × percent body weight/((height)^2^(m)^2^ × 22). Subsequently, the functional oral intake scale (FOIS) and Barthel index (BI) before admission were recorded as basic characteristics. These assessments before admission were collected through interviews with patients, family members, primary caregivers, or from summaries provided by the facility or care manager. The FOIS [16] was used for assessing oral intake and comprises a 7-point (1-7) ordinal scale; the highest value indicates normal swallowing ability, whereas the lowest denotes the need for tube feeding, with no oral intake. The BI [17] comprises ten items that evaluate a patient’s ability in terms of feeding, grooming, bathing, toilet use, dressing, walking, transfers, and climbing stairs, as well as fecal and/or urinary incontinence. The index is calculated by adding 5, 10, or 15 points for the presence of each item (final score, 0-100 points).

During hospitalization, the following treatment details and progress were recorded: the time of physical, occupational, and swallowing therapy; the day of the first physical therapy; the day of early sitting by a physical therapist; admission to the intensive care unit (ICU) or high care unit (HCU); noninvasive or invasive mechanical ventilator; type of antibiotic; and length of hospital stay.

Definition of early sitting

Early sitting was defined as the mobilization was categorized as the ICU Mobility Scale (IMS) category 3, within a week of admission. The IMS [18] offers a rapid and straightforward bedside method of measuring the mobility of patients with critical illness. Sitting was in IMS category 3, which may be assisted by staff but involves actively sitting over the side of the bed with some trunk control. The decision of first sitting was performed considering the initiation criteria and eyeball test [19], according to the initiation criteria in the early mobilization and active exercise in early phase in ICU proposed in the expert consensus of the Japanese Society of Intensive Care Medicine [20] and Guidelines for Safety Management and Promotion developed by the Clinical Practice Guidelines Committee of the Japanese Association of Rehabilitation Medicine [21]. The eyeball test is a subjective assessment performed by physical therapists to determine if a patient is ready to sit, based on clinical intuition and observations of the patient’s facial expressions and behavioral reactions [19]. The physical therapists involved in this study were licensed professionals working full-time in the hospital. They had two to 17 years of clinical experience and were assigned to the acute care and respiratory/internal medicine wards. The important decision of the first sitting, based on both objective and subjective assessments, was always made by two or more physical therapists, taking into account that the decision was not influenced by differences in their experience.

Statistical analyses 

Data were expressed as medians (25th-75th percentile) or percentages, as appropriate. Differences in continuous variables were compared using the Wilcoxon rank-sum test, whereas categorical variables were compared using the chi-squared test. The main analysis employed multivariate logistic regression with in-hospital mortality as the dependent variable. The analysis, which accounts for multicollinearity, was conducted using several models that included potential confounders identified in previous studies instead of analyzing all variables in one model. Furthermore, all multivariate logistic regression models were separately conducted, initially excluding and subsequently including the factor of early sitting or not.

Age, sex, and BMI were included as covariates in all models. Model 1 was additionally adjusted for A-DROP, model 2 for CCI, model 3 for GNRI, and model 4 for BI before admission. The predictive accuracy of the prognostic factors was assessed using the area under the curve (AUC). To determine the priority of early sitting or not, and each factor used in the logistic regression analysis, the classification and regression tree (CART) analysis was performed as a sub-analysis. For the CART analysis, eight variables were included as predictors: age, sex, BMI, A-DROP, CCI, GNRI, BI before admission, and early sitting or not. The CART analysis was configured as follows: complexity parameter, 0.01; maximum depth of the tree diagram, 3; and minimum node size, 10. The cutoff value for the day of sitting for nonsurvivors was determined using the receiver operating characteristic (ROC) curves and the AUC. To evaluate the contribution of *early sitting* to the discriminatory performance of each logistic regression model, we compared the AUC of each model with and without the inclusion of this variable using DeLong’s test for correlated ROC curves. The optimal cutoff point was determined using the highest value of Youden’s index.

All statistical analyses were performed using EZR version 4.3.3 (Saitama Medical Center, Jichi Medical University, Saitama, Japan). The CART analysis was performed using the rpart package in R (version 4.3.2; R Foundation for Statistical Computing, Vienna, Austria). For all tests, the significance level was 0.05; however, no prespecified approach was used for multiplicity correction. Therefore, the results of the statistical analysis were not adjusted for multiplicity and should therefore be interpreted with caution.

## Results

**Figure 1:**
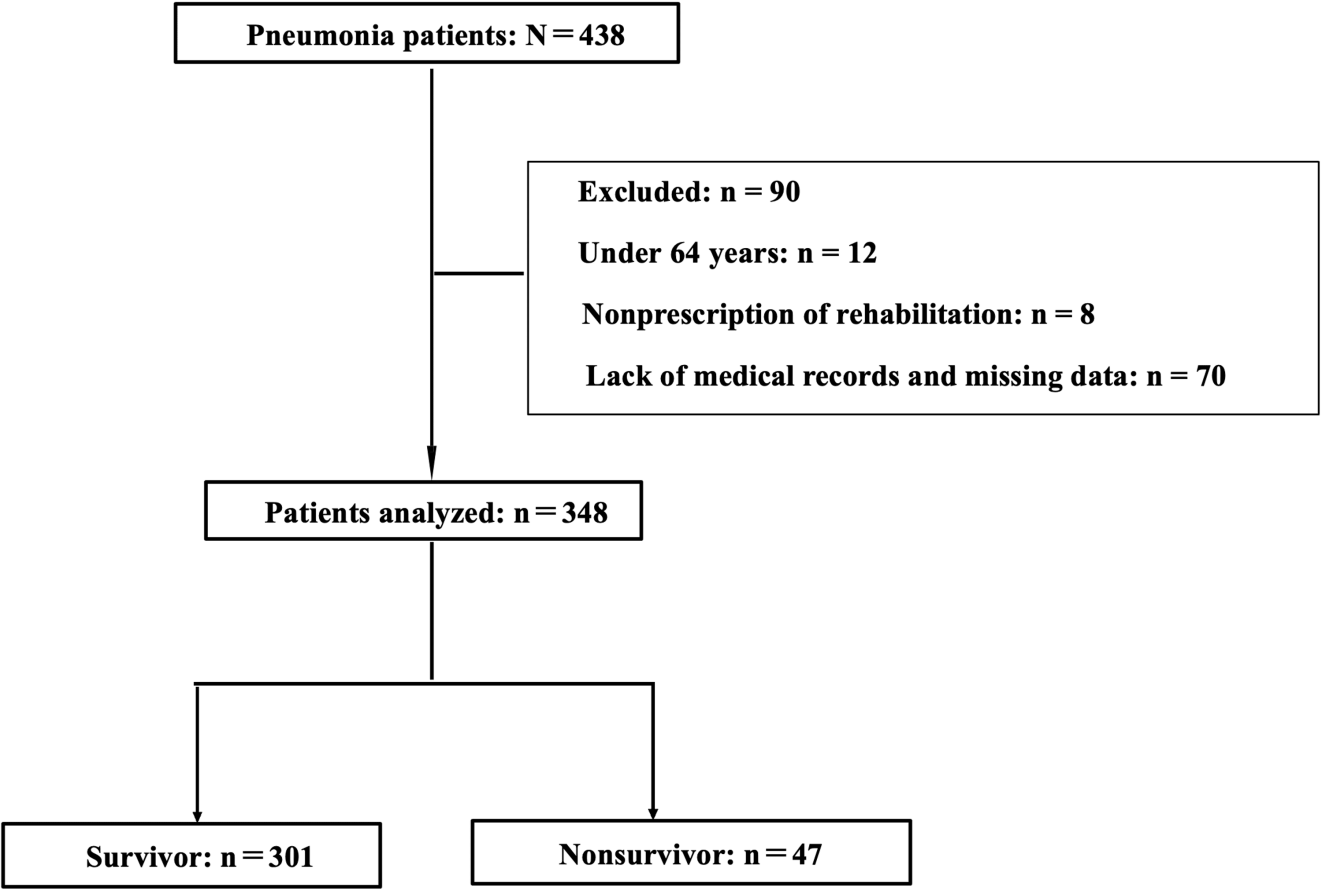
Study participation and exclusion flowchart.

During the study period, 438 patients with pneumonia were noted, of whom 90 met the exclusion criteria. Finally, a total of 348 patients were included in the analysis. Among them, 47 (13.5%) and 301 (86.5%) patients were nonsurvivors and survivors, respectively (Figure 1).

Participant characteristics 

The patient characteristics and differences between the nonsurvivor and survivor groups are shown in Table 1. The median age of patients was 84 years (interquartile range (IQR), 79-90), and 189 (54.3%) patients were males. The nonsurvivor group exhibited a significantly higher A-DROP score than the survivor group (median, 4; IQR, 3-4 vs. median, 2; IQR, 2-3; *P* < 0.001). The nonsurvivor group also had a significantly higher CCI (median, 3; IQR, 2-4 vs. median, 2; IQR, 2-3; *P* = 0.008). Regarding nutritional status, BMI did not show a significant difference. The nonsurvivor group had a significantly lower GNRI than the survivor group (median, 71.8; IQR, 66.0-79.2 vs. median, 79.3; IQR, 71.6-87.5; *P* < 0.001). Before admission, both the BI (median, 5; IQR, 0-20 vs. median, 10; IQR, 0-50; *P* = 0.016) and FOIS (median, 5; IQR, 2.5-6 vs. median, 5; IQR, 4-7; *P* = 0.018) were lower in the nonsurvivor group than those in the survivor group. Regarding the progress of hospitalization, 326 patients (93.7%) performed sitting during hospitalization. Among them, 307 patients (88.2%) achieved early sitting. The day of sitting in the nonsurvivor group was significantly later than in the survivor group (median, 5 days; IQR, 3-7.5 vs. median, 2 days; IQR, 2-3; *P* < 0.001). The nonsurvivor group had a greater number of patients admitted to the ICU or HCU than the survivor group (42.6% vs. 9.6%, *P* < 0.001). The nonsurvivor group demonstrated a higher percentage of patients on mechanical ventilation than the survivor group (10.6% vs. 3.7%, *P* = 0.033). No significant differences in the type of antibiotic and length of hospital stay were observed between the two groups.

**Table 1:** Participant characteristics and differences between the nonsurvivor and survivor groups. Data are presented as medians (interquartile ranges) or *n* (%). CAP, community-acquired pneumonia; HCAP, healthcare-associated pneumonia; WBC, white blood cell count; CRP, C-reactive protein; Alb, serum albumin; BUN, blood urea nitrogen; CCI, Charlson comorbidity index; BMI, body mass index; FOIS, functional oral intake scale; GNRI, geriatric nutritional risk index; BI, Barthel index; ICU, intensive care unit; HCU, high care unit; SBT/ABPC, sulbactam/ampicillin; CMZ, cefmetazole; TAZ/PIPC, tazobactam/piperacillin; CTRX, ceftriaxone; LVFX, levofloxacin; IPM/CS, imipenem/cilastatin; DPRM, doripenem; CEZ, cefazolin; MINO, minocycline; CFPM, cefepime

	Total (*N* = 348)	Survivor (*n* = 301)	Nonsurvivor (*n* = 47)	*P*-value
Age, years	84 (79-90)	84 (78-90)	86 (81-90)	0.319
Male sex, *n*	189 (54.3)	155 (51.5)	34 (72.3)	0.012 (df = 1, *V* = 0.13)
Characteristics of pneumonia, *n*				0.138 (df = 1, *V* = 0.07)
CAP	134 (38.5)	121 (40.2)	13 (27.7)	
HCAP	214 (61.5)	180 (59.8)	34 (72.3)	
Laboratory date				
WBC (μL)	11,640 (8,877-15,010)	11,630 (9,070-14,940)	11,650 (8,260-15,940)	0.506
CRP (mg/dL)	12.1 (7.5-18.1)	11.6 (7.0-16.5)	15.6 (12.6-24.0)	<0.001
Alb (g/dL)	3.0 (2.6-3.3)	3.0 (2.6-3.3)	2.6 (2.3-3.1)	<0.001
BUN (mg/dL)	21.6 (16.3-33.0)	20.5 (15.9-19.3)	28.8 (21.4-37.2)	0.001
A-DROP	3 (2-3)	2 (2-3)	4 (3-4)	<0.001
Comorbidity, *n*				
Cerebrovascular disease	175 (50.3)	156 (51.8)	19 (40.4)	0.146 (df = 1, *V* = 0.06)
Pulmonary disease	55 (15.8)	39 (13.0)	16 (34.0)	<0.001 (df = 1, *V* = 0.18)
Cardiovascular disease	60 (17.2)	48 (15.9)	12 (25.5)	0.158 (df = 1, *V* = 0.07)
Liver disease	4 (1.1)	4 (1.3)	0	0.952 (df = 1, *V* < 0.01)
Kidney disease	25 (7.2)	16 (5.3)	9 (19.1)	<0.001 (df = 1, *V* = 0.16)
Diabetes mellitus	87 (25.0)	76 (25.2)	11 (23.4)	0.927 (df = 1, V < 0.01)
Cancer	29 (8.3)	21 (7.0)	8 (17.0)	0.042 (df = 1, *V* = 0.10)
CCI, points	2 (2-3)	2 (2-3)	3 (2-4)	0.008
Nutritional status				
BMI (kg/m^2^)	18.4 (16.4-20.8)	18.5 (16.5-20.9)	17.8 (15.5-19.1)	0.059
Oral function				
FOIS before admission	5 (4-7)	5 (4-7)	5 (2.5-6)	0.018
GNRI	78.7 (70.7-87.1)	79.3 (71.6-87.5)	71.8 (66.0-79.2)	<0.001
BI before admission	0 (0-10)	10 (0-50)	5 (0-20)	0.016
Progress of rehabilitation				
Day of physical therapy	2 (2-2)	2 (2-2)	2 (2-3)	<0.001
Sitting during hospitalization	326 (93.7)	295 (98.0)	31 (66.0)	<0.001 (df = 1, *V* = 0.43)
Early sitting	307 (88.2)	284 (94.3)	23 (48.9)	<0.001 (df = 1, *V* = 0.46)
Day of sitting	2 (2-4)	2 (2-3)	5 (3-7.5)	<0.001
Time of rehabilitation per day (minutes)	46 (36-54)	48 (38-56)	28 (20-36)	<0.001
Admission to ICU/HCU (*n*)	46 (13.2)	29 (9.6)	20 (42.6)	<0.001 (df = 1, *V* = 0.31)
Mechanical ventilator (*n*)	16 (4.6)	11 (3.7)	5 (10.6)	0.033 (df = 1, *V* = 0.09)
Type of antibiotic (*n*)				0.179 (df = 9, *V* = 0.19)
SBT/ABPC	224 (64.4)	192 (63.8)	32 (68.0)	
CMZ	20 (5.7)	18 (6.0)	2 (4.3)	
TAZ/PIPC	12 (3.4)	10 (3.3)	2 (4.3)	
CTRX	58 (16.7)	52 (17.3)	6 (12.8)	
LVFX	10 (2.9)	9 (3.0)	1 (2.1)	
IPM/CS	4 (1.2)	4 (1.3)	0	
DPRM	8 (2.3)	4 (1.3)	4 (8.5)	
CEZ	5 (1.4)	5 (1.7)	0	
MINO	1 (0.3)	1 (0.3)	0	
CFPM	5 (1.4)	5 (1.7)	0	
No antibiotic	1 (0.3)	1 (0.3)	0	
Length of hospital stay (day)	18 (11-29)	18 (11-28)	19 (11.5-29.5)	0.964

In-hospital mortality prediction 

**Figure 2:**
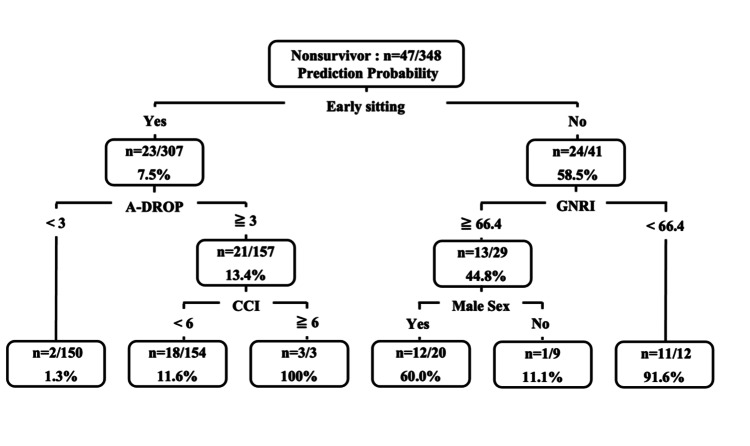
Decision tree analysis for predicting in-hospital mortality. GNRI, geriatric nutritional risk index; CCI, Charlson comorbidity index

The results of the multivariable logistic regression analysis of factors contributing to in-hospital mortality are presented in Table 2. Early sitting was a significant independent factor in all models. In model 1, results showed that male sex (odds ratio [OR] = 2.74, 95% confidence interval [CI], 1.17-6.43, *P* = 0.020), A-DROP (OR = 2.37, 95% CI, 1.56-3.60, *P* < 0.001) and early sitting (OR = 0.09, 95% CI, 0.04-0.22, *P* < 0.001) were independent factors. In model 2, male sex (OR = 2.85, 95% CI, 1.23-6.60, P = 0.014), CCI (OR = 1.28, 95% CI, 1.02-1.61, *P* = 0.033), and early sitting (OR = 0.06, 95% CI, 0.02-0.13, *P* < 0.001) were independent factors. Model 3 showed that male sex (OR = 2.92, 95% CI, 1.28-6.69, *P* = 0.011) and early sitting (OR = 0.07, 95% CI, 0.03-0.15, *P* < 0.001) were independent factors. Model 4 demonstrated that male sex (OR = 3.23, 95% CI, 1.41-7.38, *P* < 0.001) and early sitting (OR = 0.06, 95% CI, 0.03-0.14, *P* < 0.001) were independent factors. The AUC for each model increased when early sitting was added as a factor (model 1, 0.819 vs. 0.874, *P* = 0.005; model 2, 0.713 vs. 0.831, *P* < 0.001; model 3, 0.733 vs. 0.820, *P* < 0.001; model 4, 0.709 vs. 0.817, *P* < 0.001). The CART analysis showed that early sitting was identified as the parent node, and 24 patients (58.5%) of the participants who did not perform early sitting were classified as nonsurvivors (Figure 2).

**Table 2:** Multivariable logistic regression analysis of factors contributing to in-hospital mortality BMI, body mass index; CCI, Charlson comorbidity index; GNRI, geriatric nutritional risk index; BI, Barthel index; OR, odds ratio; CI, confidence interval

	OR	95% CI	*P*-value	AUC	OR	95% CI	*P*-value	AUC
Model 1				0.819				0.874
Age (years)	1.03	0.98-1.08	0.235		1.03	0.97-1.08	0.329	
Male sex	3.07	1.42-6.67	0.004		2.74	1.17-6.43	0.020	
BMI	0.94	0.84-1.06	0.318		0.95	0.84-1.08	0.480	
A-DROP	2.90	1.98-4.24	<0.001		2.37	1.56-3.60	<0.001	
Early sitting					0.09	0.04-0.22	<0.001	
Model 2				0.713				0.831
Age, years	1.05	1.00-1.09	0.047		1.05	0.99-1.10	0.068	
Male sex	3.07	1.46-6.44	0.003		2.85	1.23-6.60	0.014	
BMI	0.89	0.80-1.00	0.059		0.93	0.82-1.06	0.285	
CCI	1.27	1.04-1.56	0.020		1.28	1.02-1.61	0.033	
Early sitting					0.06	0.02-0.13	<0.001	
Model 3				0.733				0.820
Age, years	1.04	0.99-1.09	0.071		1.04	0.99-1.10	0.089	
Male sex	2.93	1.41-6.08	0.003		2.92	1.28-6.69	0.011	
BMI	1.04	0.89-1.22	0.591		1.01	0.85-1.19	0.940	
GNRI	0.94	0.90-0.98	0.003		0.96	0.92-1.01	0.125	
Early sitting					0.07	0.03-0.15	<0.001	
Model 4				0.709				0.817
Age, years	1.05	1.00-1.09	0.052		1.05	0.99-1.10	0.066	
Male sex	3.50	1.68-7.31	<0.001		3.23	1.41-7.38	<0.001	
BMI	0.92	0.81-1.04	0.172		0.93	0.82-1.06	0.285	
BI before admission	0.98	0.97-1.00	0.074		0.99	0.98-1.01	0.479	
Early sitting					0.06	0.03-0.14	<0.001	

**Figure 3:**
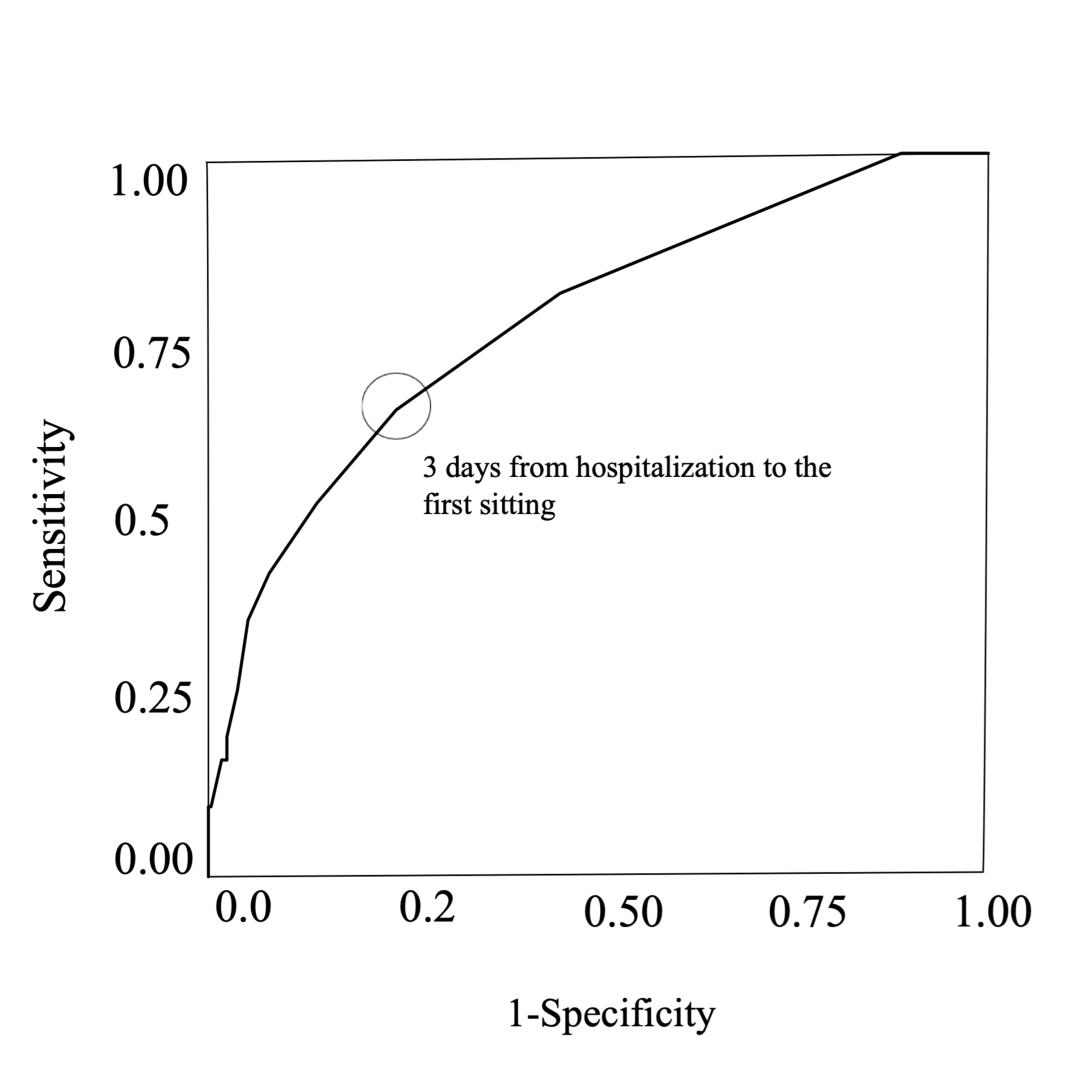
Receiver operating characteristic curve analysis for in-hospital mortality. Positive is defined as in-hospital mortality. The cutoff point of the day from hospitalization to the first sitting is 3 days: sensitivity, 0.65; specificity, 0.76; area under the curve, 0.76.

In the ROC curve of the day of sitting for in-hospital mortality (Figure 3), 22 patients who were unable to sit during hospitalization were excluded. The day of sitting, used for predicting in-hospital mortality, was evaluated in 326 patients (survivor group, *n* = 295; nonsurvivor group, *n* = 31). The AUC for the day of sitting was 0.76. The cutoff value for the day from hospitalization to the first sitting for determining in-hospital mortality was day 3 (sensitivity, 64.5; specificity, 75.9).

## Discussion

We investigated whether early sitting improves the accuracy of predicting in-hospital mortality in older adult patients with pneumonia. The results revealed that early sitting by the physical therapist was a predictor of in-hospital mortality in older adult patients with pneumonia in all models, along with sex, A-DROP, and CCI [4-7], which are predictors of in-hospital mortality in patients with pneumonia. Furthermore, in all models, the accuracy of prognosis prediction improved when early sitting by physical therapists was included. These results suggest that adding early sitting to admission-based factors may enhance the prediction of in-hospital mortality in older adult patients with pneumonia.

Early mobilization may help prevent pneumonia progression following hospitalization and is frequently integrated into physical therapy interventions for patients with pneumonia [12,22]. Therefore, it is possible that achieving early mobilization improved the day from hospitalization to the first sitting in the survivor group. On the other hand, such studies often focus on the effect of achieving early mobilization, but it may also be important to decide whether the condition is amenable to an attempt.

This study suggests that the decision of physical therapists to initiate sitting during early hospitalization can predict in-hospital mortality in older adult patients with pneumonia. Several guidelines have provided criteria for the decision to initiate sitting [23,24]. Along with the attending physician’s order, the physical therapist decides to initiate sitting using objective measures, including blood pressure, level of consciousness, and transcutaneous oxygen saturation, which are items in the initiation criteria for early mobilization. Notably, these items are also included in the A-DROP, indicating an overlap between the initiation criteria and A-DROP. Thus, early sitting by physical therapists may indicate disease severity following hospitalization. In other words, even if the disease severity was high at admission, patients who received early sitting by physical therapists likely satisfied the criteria for sitting because they exhibited rapid improvement in their medical condition owing to antimicrobial agents and other medical treatments such as intravenous fluids and oxygen therapy. Occasionally, older adult patients with pneumonia are less likely to exhibit typical respiratory symptoms and may present with symptoms, including loss of appetite and decreased activity [12]. Furthermore, this study included patients who were unable to report their symptoms before hospitalization owing to impaired consciousness or dementia. Consequently, the physical therapists in this study decided to initiate sitting using both objective and subjective assessments, including impressions and intuition gained from participants’ facial expressions and behavioral reactions. Subjective assessment is frequently employed in clinical practice owing to its ease and speed of administration. It is reportedly useful for predicting mortality in patients with aortic stenosis [19] and functional prognosis in patients with musculoskeletal pain [25]. In contrast, one study described that subjective assessment was less useful than objective assessment [26]. Therefore, considering the severity and impact of the disease, a previous study suggested performing a comprehensive assessment, encompassing subjective and objective assessments, for predicting prognosis [27]. In the present study, the physical therapists decided for patients to sit based on subjective and objective assessments. The decision likely reflected the overall index of the patient’s condition during the early post-hospitalization period. Therefore, early sitting by physical therapists during hospitalization was identified as a predictor of in-hospital mortality.

Notably, in all models, the accuracy of prognosis prediction increased when early sitting by the physical therapist was integrated into each model. Based on this finding, a more accurate prediction of in-hospital mortality may be achieved by incorporating the physical therapist’s decision to perform early sitting after admission, in addition to factors predicting in-hospital mortality at admission. The Japanese Respiratory Society guidelines for the treatment of pneumonia in adults have indicated that medical professionals should appropriately assess patients with terminal pneumonia; share assessment results with the patients, their family, and caregivers; support decision making; and provide multidisciplinary care [12]. Therefore, a more accurate predictor of in-hospital mortality may not only serve as a criterion for physical therapists to determine the optimal treatment but also assist patients and their families in making decisions on treatment.

This study had several limitations. First, as this study lacked external validity owing to its single-center design and the heterogeneity in the population, which was characterized by markedly older age, low BMI, and low activities of daily living (ADLs), the results should be interpreted with caution. Second, as this was a retrospective cohort study, the data collected were limited. The possibility of confounding or other relevant factors, such as the amount and content of rehabilitation performed from sitting initiation until discharge, cannot be ruled out. Third, although we enrolled consecutive pneumonia patients who were referred for rehabilitation, patients not prescribed rehabilitation were excluded; this restriction may have introduced selection bias. Fourth, although the decision to initiate sitting was made collaboratively by physical therapists, it was based in part on subjective clinical judgment, which may differ depending on each therapist’s individual experience and skill level. Fifth, although this study focused on early sitting, it remains unclear whether the clinical decision to initiate sitting or the actual act of sitting is more important for prognosis.

## Conclusions

We investigated the prognostic factors for in-hospital mortality in older adult patients with pneumonia who were prescribed rehabilitation. This study revealed that factors at admission, as in previous studies, were the predictors of in-hospital mortality, and that adding the factor of early sitting may increase the accuracy of predicting in-hospital mortality.

Future research should be conducted as a multicenter study to ensure external validity. Additionally, it is necessary to establish an algorithm using decision tree analysis and determine a cutoff value with high sensitivity and specificity.
